# Evaluation of Federal Policy Changes to the Hospice Benefit and Use of Hospice for Persons With ADRD

**DOI:** 10.1001/jamahealthforum.2022.0900

**Published:** 2022-05-06

**Authors:** Kan Z. Gianattasio, Ali Moghtaderi, Dale Lupu, Christina Prather, Melinda C. Power

**Affiliations:** 1 Department of Health Policy and Management, George Washington University Milken Institute School of Public Health, Washington, DC; 2 Department of Health Care Evaluation, NORC at the University of Chicago, Bethesda, Maryland; 3 George Washington University School of Nursing, Washington, DC; 4 Division of Geriatric and Palliative Medicine, George Washington School of Medicine and Health Sciences, Washington, DC; 5 Department of Epidemiology, George Washington University Milken Institute School of Public Health, Washington, DC

## Abstract

**Importance:**

Hospice is an important end-of-life service for patients with Alzheimer disease and related dementias (ADRD).

**Objective:**

To determine whether hospice use among patients with ADRD changed in association with recent policies aimed at reducing hospice misuse and long hospice stays, an outcome that may have disproportionately affected patients with ADRD because of their lengthy end-of-life trajectories.

**Design, Setting, and Participants:**

This observational cross-sectional study used Medicare hospice claims data from Medicare hospice episodes of care beginning between July 2008 and December 2019 among Medicare hospice beneficiaries 65 years or older at time of enrollment. Data analysis was conducted between September 2019 and June 2021.

**Exposures:**

The 2014 Improving Medicare Post-Acute Care Transformation (IMPACT) Act, which systematized audits of hospices with a high proportion of long stays, and the 2016 2-tier payment system, which reduced daily reimbursement rates after 60 days.

**Main Outcomes and Measures:**

Monthly percentage of (1) new patient enrollees, (2) patient census, and (3) care days provided to patients with an ADRD code.

**Results:**

The sample included 11 124 992 unique hospice episodes between 2008 and 2019; mean (SD) patient enrollment age ranged from 82.0 (8.2) years to 82.8 (8.7) years; the percentage of male patients ranged from 40.5% to 42.7%, and the percentage of Black, Hispanic, and White patients ranged from 7.7% to 8.2%, 1.5% to 2.0%, and 86.2% to 88.8%, respectively, across years. The percentage of new enrollees with an ADRD code dropped significantly during the months of IMPACT passage (−1.42 percentage points; 95% CI, −2.13 to −0.71) and implementation (−1.98 percentage points; 95% CI, −2.70 to −1.26) but rose again during the following months. While no significant changes were observed at the time of 2-tier payment implementation (0.15 percentage points; 95% CI, −0.21 to 0.51), the average rate of increase during the subsequent period was slower (0.01 percentage points per month; 95% CI, 0-0.02) than in earlier periods (0.05; 95% CI, 0.04-0.06 during the baseline period). Similar patterns were observed for the percentage of patient census and care days provided to patients with an ADRD code.

**Conclusions and Relevance:**

The results of this cross-sectional study of Medicare hospice claims data suggested that recent Medicare policies were associated with immediate and lasting reductions in the share of patients receiving hospice care with an ADRD code compared with expectations from preimplementation trends. Future research should examine mechanisms through which hospices enacted change and consequences for quality of care.

## Key Points

**Question:**

Did hospice use for persons with Alzheimer disease and related dementias (ADRD) change between 2008 and 2019 in conjunction with Medicare policy changes that aimed to reduce long hospice stays?

**Findings:**

In this cross-sectional study of Medicare claims data from 11 124 992 unique hospice episodes, there were immediate declines in the share of patients receiving hospice care with ADRD and a slower growth in use of hospice care among patients with ADRD after implementation of the US Improving Medicare Post-Acute Care Transformation Act and the 2-tier payment system compared with prepolicy trends.

**Meaning:**

The study results suggest that reduced utilization of hospice by patients with ADRD following these policy changes may be negatively associated with end-of-life experience and outcomes for persons with ADRD.

## Teaser

This cross-sectional study examines the association of hospice use among patients with Alzheimer disease and related dementias with the passage of the US IMPACT Act and implementation of the 2-tier payment system.

## Introduction

Hospice comprehensively supports quality of life for patients with terminal illness and their family caregivers.^1^ Hospice is the preferred approach to care and is particularly beneficial for many patients with Alzheimer disease and related dementias (ADRD) given the disease’s prolonged period of decline, uniquely burdensome symptoms and resulting dependencies, and lack of proven disease-modifying treatments.^2,3,4,5,6,7,8,9,10^ Patients with ADRD typically have longer hospice stays than patients with other diagnoses^11,12^ and represent a large and growing hospice patient population,^13,14^ although some of the observed growth may reflect coding changes. Notably, a large increase in patients with ADRD receiving hospice care from 2012 to 2013^11,15^ likely reflects a shift toward greater use of dementia codes following notice to exclude failure to thrive (FTT) and debility as qualifying hospice diagnoses.^16^

Medicare covers hospice for those with a 6-month or shorter prognosis. As the primary insurer of patients with ADRD,^17,18^ Medicare policies appreciably affect eligibility, access, and use of hospice by patients with ADRD. Using the prior flat payment system, patients with longer hospice stays, including patients with ADRD, generated greater profits for hospices given that average daily costs of providing care are highest during the first and last weeks of a stay.^19,20^ This raised concerns about fraudulent/inappropriate patient selection practices that favored long-stay patients, including patients with ADRD. In response, 2 Medicare hospice policy changes were introduced to discourage hospice selection for long-stay patients through increased regulatory scrutiny and reduced profitability.^19,21,22,23,24,25^ First, the 2014 Improving Medicare Post-Acute Care Transformation (IMPACT) Act included systemization of audits that targeted hospices with high proportion of patient stays longer than 180 days among other changes, including tightened hospice provider survey frequencies and adjustments to the aggregate cap calculation.^26^ Second, the introduction of the 2016 2-tier payment system reduced routine home care reimbursement after 60 days.^22^ Given that patients with ADRD have the highest likelihood of hospice stays that are longer than 180 days and often have compromised capacity for choice and self-advocacy, patients with ADRD may have been uniquely affected by these policies.^7,11,12^

Thus, the goal of this study was to describe trends in the share of patients with ADRD in hospice before, during, and after implementation of the IMPACT Act and the 2-tier payment system while accounting for changes to coding practices because of the FTT/debility exclusion. We hypothesize that these policies decreased the share of patients with ADRD, with more pronounced effects in for-profit hospices.

## Methods

This is a cross-sectional study of Medicare claims data. The study followed all relevant Strengthening the Reporting of Observational Studies in Epidemiology (STROBE) reporting guidelines and was approved by the George Washington University institutional review board. Because we used claims data, there was no interaction with patients, and therefore no informed consent procedures were required.

### Data Sources and Study Sample

We used the 2008 to 2019 100% limited data set of Medicare hospice claims. These data contain *International Classification of Diseases, Ninth Revision (ICD-9)*/*ICD-10* codes indicating the principal and contributing terminal conditions of patients, claim dates, levels, and sites of service. We linked these to (1) the master beneficiary summary/denominator files to obtain basic patient demographic characteristics (age, sex, and race and ethnicity [originating from Social Security Administration records]^27^), death date, and Medicare Advantage (MA) enrollment; and (2) provider of service files to obtain data on hospice ownership, affiliations, and employee statistics. Additionally, we used claims files to construct monthly hospice-level demographic characteristics of new enrollees and the proportion of care days provided across levels (eg, routine home care) and sites of care (eg, a skilled nursing facility). Finally, we linked US Census data and the Area Health Resources Files to obtain county-level characteristics, including total population, demographic distributions (sex, race and ethnicity, and age), number of physicians, average household income, percentage living with poverty, and rural-urban continuum classification.

The US Centers of Medicare & Medicaid Services hospice billing guidelines require a separate claim for every calendar month of enrollment. We constructed care episodes by combining consecutive claims with identical beneficiary and hospice organization IDs, and we identified unique episodes starting with any new enrollment, any reenrollment following discharge, or any new claims following a break in claims of more than 31 days. Starting with episodes beginning between July 2008 and December 2019 (n = 13 812 688), we excluded episodes with missing hospice-level or county-level covariates (11.5%); episodes of beneficiaries younger than 65 years or with missing demographic characteristics (5.8%); episodes with claims billed using nonsequential dates, duplicate dates, or dates inconsistent with master beneficiary summary death dates (0.7%); and episodes violating the requirement for calendar-month claims (1.5%). The final sample included 11 124 922 episodes (eTable 1 in the Supplement). There were no appreciable changes in patient-level or hospice-level characteristics after exclusions (eTable 2 in the Supplement).

### Outcomes

We identified patients with ADRD based on a principal or secondary diagnosis of ADRD using *ICD-9* and *ICD-10* codes (eTable 3 in the Supplement^28^). We constructed 3 hospice-level outcomes: (1) monthly percentage of new patient enrollees with ADRD, (2) monthly percentage of patient census (ie, count of patients receiving at least 1 day of care during a given month) with ADRD, and (3) monthly percentage of care days provided to patients with ADRD. We referred to the percentage of patient census and care days as *caseload outcomes*.

### Statistical Analyses

We modelled the trends in each outcome over time and in associated with the FTT/debility exclusion notice in May 2013 (to account for code shifting), IMPACT Act passage in September 2014, IMPACT Act implementation in October 2014, and 2-tier payment implementation in January 2016. We used (1) indicator terms to estimate immediate changes in outcomes at the time of each policy event and (2) linear time splines to estimate slopes in outcome trends during each period demarcated by the events described previously. While other policy changes occurred during the early years of the analytical period (notably, the face-to-face recertification requirement [2011] and introduction of Program for Evaluating Payment Patterns Electronic Report reports [2012]), we did not account for them given lack of evidence of associations with overall trends in the outcomes. We adjusted for month fixed effects to account for seasonality and time-varying hospice-level and county-level characteristics (eTable 4 in the Supplement) and adjusted for hospice fixed effects to control for non–time-varying hospice characteristics and estimate within-hospice variations across time.^29^ Model specification details are provided in eAppendix 1 in the Supplement.

Given the insubstantial association of covariate adjustment with the coefficients of interest, we ran and plotted results from minimally adjusted (adjusting only for month and hospice fixed effects) versions of the model and minimally adjusted descriptive models parameterizing time as a series of indicator variables for each postpolicy month to visualize trends over time and consider the possibility of nonlinear trends. We also conducted sensitivity analyses to assess robustness of findings to (1) variations in patients with ADRD and hospice episode identification strategies; (2) exclusion of data from government-owned hospices, beneficiaries with MA coverage, hospices newly entering or exiting the market, and 2008 to 2009 claims for which only the date of service quarter indicators were available; and (3) differences between hospices with a low vs high baseline proportion of care provided to patients in institutionalized settings (hereafter referred to as low vs high institutionalization hospices). Further details of the sensitivity analyses are available in eAppendix 2 in the Supplement.

Finally, we ran stratified analyses to evaluate differential policy effects by hospice ownership (for-profit vs nonprofit/government-owned) and tested for effect modification by adding interaction terms of ownership with the time spline and policy indicator terms. Because hospice ownership is non–time-varying for most hospices, we excluded hospice fixed effects in these models. Analyses were conducted using SAS, version 9.4 (SAS Institute), and Stata, version 15 (StataCorp), between September 2019 and June 2021.

## Results

Hospice episode characteristics are summarized in Table 1. There was a steady rise in total care episodes provided (802 101 to 1 164 239), MA coverage (24% to 39%), share of patients with an ADRD code (16% to 25%), and care episodes provided at for-profit institutions (44% to 53%) between 2009 and 2019. eTable 5 in the Supplement provides summary characteristics of hospices in operation each year; the proportion of for-profit institutions rose substantially from 2009 to 2019 (55% to 75%).

**Table 1.** Summary of Patient Characteristics and Hospice Characteristics Across Eligible Hospice Episodes

Characteristic	Patients, %											
	2008^a^	2009	2010	2011	2012	2013	2014	2015	2016	2017	2018	2019
No.	383 148	802 101	839 906	873 320	909 635	944 745	949 582	992 004	1 033 985	1 099 699	1 132 558	1 164 239
Patient age at enrollment, mean (SD), y	82.0 (8.2)	82.4 (8.2)	82.5 (8.3)	82.6 (8.3)	82.7 (8.4)	82.7 (8.5)	82.7 (8.5)	82.8 (8.6)	82.8 (8.6)	82.8 (8.7)	82.8 (8.7)	82.7 (8.7)
Female patients	59.5	59.2	59.1	59.1	58.241.2	58.5	58.2	58.1	57.9	57.7	57.5	57.3
Male patients	40.5	40.8	40.9	40.9	41.2	41.5	41.8	41.9	42.1	42.3	42.5	42.7
Patient race and ethnicity												
Black	7.8	7.7	7.7	7.8	7.8	7.9	7.9	7.9	8.1	8.1	8.2	8.2
Hispanic	1.5	1.5	1.6	1.6	1.6	1.7	1.7	1.8	2.0	2.0	2.0	2.0
White	88.7	88.8	88.7	88.5	88.2	87.9	87.7	87.4	86.9	86.7	86.5	86.2
Other^b^	2.0	2.0	2.1	2.2	2.3	2.5	2.7	2.9	3.1	3.3	3.4	3.6
Patient had any MA coverage	24.0	24.4	24.7	25.4	26.7	28.1	3.0	32.1	33.0	35.2	37.4	39.2
Patient with ADRD	16.0	16.4	17.2	17.8	18.8	21.9	23.0	22.5	23.5	23.9	24.3	24.5
Region												
Northeast	15.9	16.0	16.2	16.9	16.7	16.5	16.3	16.5	15.9	15.8	15.3	14.6
West	18.9	19.0	19.3	19.9	19.6	20.3	20.4	21.1	21.0	20.9	20.6	20.8
South	41.3	41.2	40.6	39.6	39.5	39.4	39.7	38.8	39.4	39.4	40.1	40.3
Midwest	23.9	23.8	24.0	23.7	24.1	23.8	23.6	23.6	23.7	24.0	24.0	24.3
Rurality												
Large metropolitan are	87.3	87.4	87.5	87.6	87.5	89.1	89.2	89.2	89.4	89.3	88.8	89.0
Urban, adjacent to metropolitan area	7.5	7.5	7.5	7.5	7.6	6.6	6.5	6.5	6.4	6.5	6.7	6.5
Urban, nonadjacent to metropolitan area	4.5	4.5	4.6	4.5	4.5	3.9	3.9	3.9	3.8	3.8	4.1	4.2
Rural	0.7	0.5	0.5	0.4	0.4	0.4	0.4	0.4	0.4	0.4	0.4	0.4
Hospice ownership												
For-profit	43.5	43.7	45.5	46.9	48.7	49.6	50.5	51.3	51.5	49.7	51.5	53.2
Nonprofit	54.6	54.6	52.8	51.3	49.2	48.2	47.5	46.6	46.7	48.5	46.7	45.1
Government-owned	1.8	1.8	1.8	1.7	2.1	2.2	2.0	2.1	1.8	1.8	1.8	1.7
Freestanding	70.7	71.9	73.0	75.0	76.9	78.3	80.4	81.8	81.6	82.3	82.8	83.7

Abbreviations: ADRD, Alzheimer disease and related dementias; MA, Medicare Advantage.

^a^ July 2008 to December 2008 only.

^b^ Includes Asian and North American Native individuals as well as those of Unknown and Other race and ethnicity.

**Figure 1.**
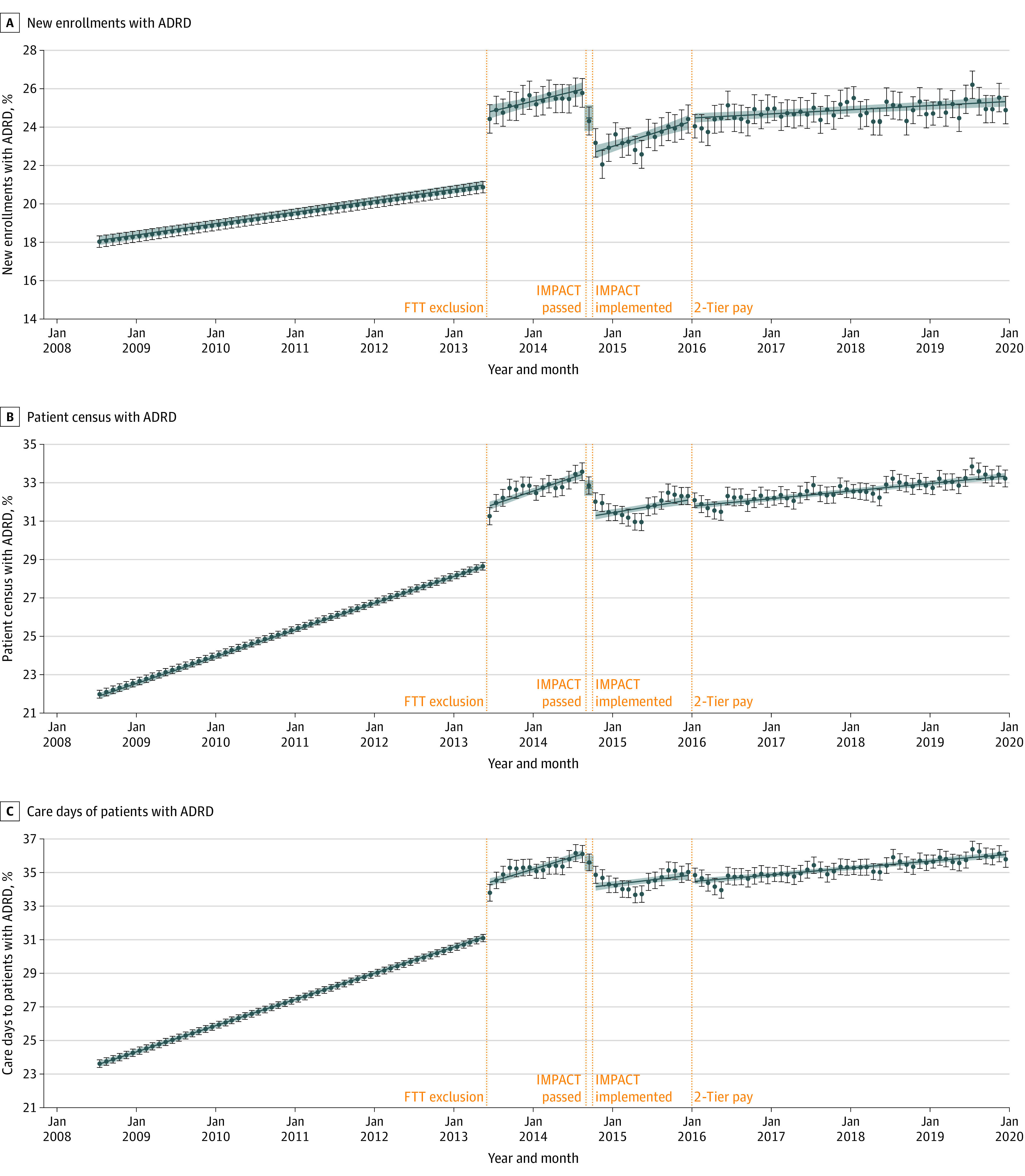
Estimated Trends in the Percentage of New Monthly Enrollees With Alzheimer Disease and Related Dementias (ADRD), Monthly Patient Census With ADRD, and Care Days Provided to Patients With ADRD From July 2008 to December 2019 We ran minimally adjusted models (adjusted for month indicators and hospice fixed effects) treating postpolicy time splines as continuous variables (as shown in Table 2), as well as minimally adjusted descriptive models treating postpolicy time splines as series of binary indicators to estimate a separate coefficient for each month. Predicted trends shown (blue lines from linear models; colored circles from descriptive models) were computed by fitting model coefficients to hospice-month observations; to allow for clearer illustration of these trends over time, we excluded seasonality effects by ignoring the month fixed effects coefficients (ie, setting them to 0) when computing predicted trends. FTT indicates failure to thrive/debility; IMPACT, Improving Medicare Post-Acute Care Transformation Act; pay, payment.

The percentage of new enrollees with ADRD increased immediately following FTT/debility exclusion notice and continued to rise during the following months, although corresponding estimates from descriptive models suggest that the rate of increase slowed slightly after 7 months (Table 2; Figure 1). The percentage of new enrollees with ADRD then dropped significantly during the months of IMPACT Act passage (−1.42 percentage points; 95% CI, −2.13 to −0.71) and implementation (−1.98 percentage points; 95% CI, −2.70 to −1.26) (Table 2; Figure 1). While it began to rise again in the following months, the descriptive models suggest a nonlinear pattern (Figure 1). Finally, there was no clear change in the percentage of new enrollees with ADRD at the time of the 2-tier payment system implementation, although the rate of increase during subsequent months was slower than before (Table 2). By 2019, the percentage of new enrollees with ADRD returned to a level similar to that observed immediately before IMPACT Act passage (Figure 1).

**Table 2.** Differences in the Trajectories of the Percentage of New Monthly Enrollees With ADRD, Monthly Patient Census With ADRD, and Care Days Provided to Patients With ADRD Coinciding With Recent Policy Changes Affecting the Medicare Hospice Benefit

Characteristic	Minimally adjusted^a^		Fully adjusted^b^	
	Estimate (95% CI)	*P* value	Estimate (95% CI)	*P* value
**Percentage of new monthly enrollees with ADRD**				
Pre-FTT/debility exclusion slope^c^	0.05 (0.04 to 0.05)	<.001	0.05 (0.04 to 0.06)	<.001
Change during FTT/debility exclusion notice	3.74 (3.36 to 4.11)	<.001	3.81 (3.43 to 4.18)	<.001
Post-FTT/debility exclusion slope^c^	0.08 (0.05 to 0.12)	<.001	0.09 (0.05 to 0.12)	<.001
Change during IMPACT Act passage	−1.49 (−2.22 to −0.76)	<.001	−1.42 (−2.13 to −0.71)	<.001
Change during IMPACT Act implementation	−1.87 (−2.61 to −1.13)	<.001	−1.98 (−2.70 to −1.26)	<.001
Post–IMPACT Act slope^c^	0.11 (0.07 to 0.15)	<.001	0.11 (0.07 to 0.15)	<.001
Change during TTP implementation	0.22 (−0.15 to 0.58)	.24	0.15 (−0.21 to 0.51)	.42
Post TTP-slope^c^	0.02 (0.01 to 0.02)	<.001	0.01 (0 to 0.02)	.002
**Percentage of monthly patient census with ADRD**				
Pre-FTT/debility exclusion slope^c^	0.12 (0.11 to 0.12)	<.001	0.12 (0.11 to 0.12)	<.001
Change during FTT/debility exclusion notice	3.05 (2.82 to 3.28)	<.001	3.03 (2.81 to 3.26)	<.001
Post-FTT/debility exclusion slope^c^	0.12 (0.09 to 0.14)	<.001	0.12 (0.10 to 0.15)	<.001
Change during IMPACT Act passage	−0.72 (−1.15 to −0.28)	.001	−0.69 (−1.12 to −0.26)	.002
Change during IMPACT Act implementation	−1.49 (−1.93 to −1.04)	<.001	−1.47 (−1.91 to −1.03)	<.001
Post–IMPACT Act slope^c^	0.06 (0.03 to 0.08)	<.001	0.05 (0.03 to 0.07)	<.001
Change during TTP implementation	−0.34 (−0.56 to−0.12)	.002	−0.36 (−0.57 to −0.15)	<.001
Post TTP-slope^c^	0.03 (0.03 to 0.04)	<.001	0.03 (0.02 to 0.03)	<.001
**Percentage of monthly care days provided to patients with ADRD**				
Pre-FTT/debility exclusion slope^c^	0.13 (0.13 to 0.13)	<.001	0.13 (0.13 to 0.14)	<.001
Change during FTT/debility exclusion notice	3.15 (2.90 to 3.40)	<.001	3.12 (2.88 to 3.37)	<.001
Post-FTT/debility exclusion slope^c^	0.12 (0.09 to 0.14)	<.001	0.13 (0.10 to 0.15)	<.001
Change during IMPACT Act passage	−0.53 (−1.01 to −0.05)	.03	−0.51 (−0.99 to −0.04)	.03
Change during IMPACT Act implementation	−1.45 (−1.94 to −0.96)	<.001	−1.40 (−1.89 to −0.92)	<.001
Post–IMPACT Act slope^c^	0.05 (0.02 to 0.07)	<.001	0.04 (0.02 to 0.06)	.001
Change during TTP implementation	−0.38 (−0.62 to −0.14)	.002	−0.40 (−0.64 to −0.17)	<.001
Post TTP-slope^c^	0.03 (0.03 to 0.04)	<.001	0.03 (0.02 to 0.03)	<.001

Abbreviations: ADRD, Alzheimer disease and related dementias; FTT, failure-to-thrive; IMPACT, Improving Medicare Post-Acute Care Transformation Act; TTP, 2-tier payment.

^a^ Adjusted for month indicators and (absorbed) hospice fixed effects.

^b^ Minimally adjusted model and hospice-level covariates (ownership [for profit vs nonprofit, free-standing vs affiliated], total registered nurses, total employees, monthly demographic distributions [age, sex, race and ethnicity] of new enrollees, monthly percentage of total care days provided at the routine home care level and continuous home care level, and monthly percentage of care days provided in the community/home setting, nonskilled long-term care setting, and skilled nursing home/inpatient setting) and county-level covariates (county population, percentage population older than 65 years and age 85 years, county sex and race and ethnicity distributions, number of active physicians, rural-urban setting, percentage living below poverty level, median household income).

^c^ Slope coefficients are direct estimates of the slope during each specified period.

Trends in the 2 caseload outcomes, percentage of total patient census with ADRD and of total care days provided to patients with ADRD, were similar (Table 2; Figure 1). Both outcomes rose following FTT/debility exclusion notice and continued to rise during subsequent months at rates similar to those observed before FTT/debility exclusion notice (Table 2). The percentage of total patient census with ADRD dropped during the months of IMPACT Act passage (−0.69 percentage points; 95% CI, −1.12 to −0.26) and implementation (−1.47 percentage points; 95% CI, −1.91 to −1.03), as did the percentage of patient care days for patients with ADRD (Table 2). This was followed by a gradual rise for both outcomes until 2-tier payment implementation, although use of linear trends masked evidence of a decline followed by recovery in both outcomes during this period (Figure 1). Following small, but significant, drops at the time of the 2-tier payment system implementation, rates of increase in both outcomes during subsequent months were slower compared with those before the FTT notice and IMPACT Act periods (Table 2).

Conclusions were similar across sensitivity analyses, with a few exceptions. When using alternate ADRD identification strategies and excluding hospices that entered or exited the market, the percentage of new enrollees with ADRD increased significantly at the time of the 2-tier payment system implementation (eTables 6 and 7 in the Supplement). Unlike in primary analyses, IMPACT ACT passage was not associated with significant reductions in the percentage of monthly care days in analyses using alternate ADRD identification strategies and was not associated with reductions in caseload outcomes when excluding MA enrollees (eTables 6 and 7 in the Supplement). Finally, high institutionalization hospices exhibited notably greater increases in all outcomes at the time of FTT/debility exclusion notice compared with the primary analyses and no further monthly changes, on average, following 2-tier payment implementation (eTable 8 in the Supplement). The percentage of new enrollees with ADRD declined more substantially at the time of IMPACT Act passage among high institutionalization hospices, but declined more substantially at the time of IMPACT Act implementation among low institutionalization hospices (eTable 8 in the Supplement).

**Figure 2.**
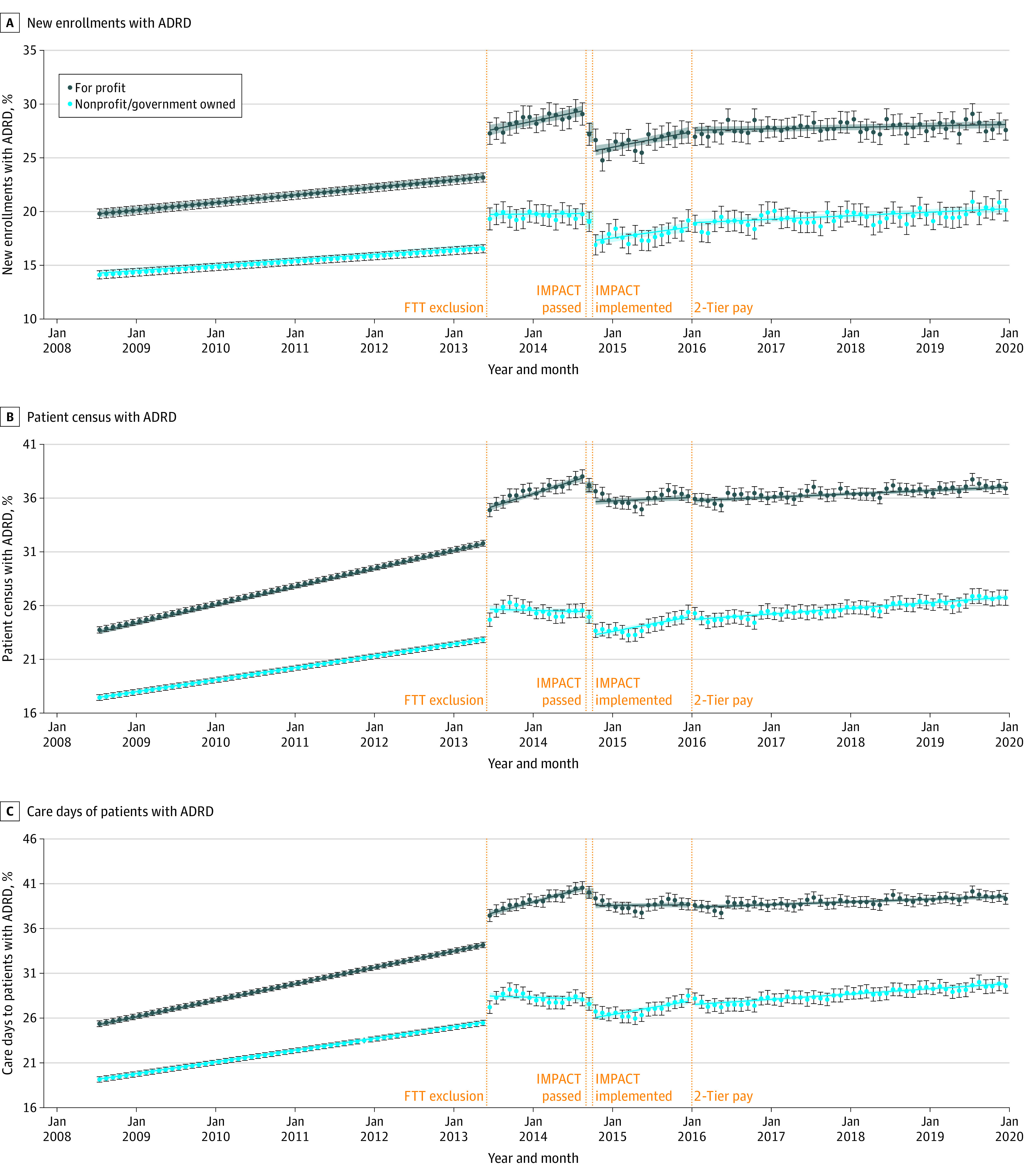
Estimated Trends in the Percentage of New Monthly Enrollees With Alzheimer Disease and Related Dementias (ADRD), Monthly Patient Census With ADRD, and Care Days Provided to Patients With ADRD by Hospice Ownership Status From July 2008 to December 2019 We ran ownership-stratified minimally adjusted models (adjusted for month indicators and hospice fixed effects) treating postpolicy time splines as continuous variables (as shown in Table 2), as well as minimally adjusted descriptive models treating postpolicy time splines as series of binary indicators to estimate a separate coefficient for each month. Predicted trends shown (blue lines from linear models; black circles from descriptive models) were computed by fitting model coefficients to hospice-month observations; to allow for clearer illustration of these trends over time, we excluded seasonality effects by ignoring the month fixed effects coefficients (ie, setting them to 0) when computing predicted trends. FTT indicates failure to thrive/debility exclusion notice; ; IMPACT, Improving Medicare Post-Acute Care Transformation Act; pay, payment.

Trends differed across for-profit and nonprofit hospices (Table 3; Figure 2). Across outcomes, the share of patients with ADRD was consistently higher in for-profit hospices than in nonprofit/government-owned hospices, with faster rates of increase in for-profit hospices before the FTT/debility exclusion notice. For-profit hospices showed a significantly greater increase in the percentage of new enrollees, but not other outcomes, at the time of the FTT/debility exclusion notice. In the months after FTT/debility exclusion but before IMPACT passage, all outcomes continued to increase in for-profit hospices but exhibited no overall change in nonprofit/government-owned hospices. Both caseload outcomes increased significantly in nonprofit/government owned hospices after IMPACT Act passage and 2-tier payment implementation.

**Table 3.** Differences in the Trajectories of the Percentage of New Monthly Enrollees With ADRD, Monthly Patient Census With ADRD, and Care Days Provided to Patients With ADRD Coinciding With Recent Policy Changes Affecting the Medicare Hospice Benefit as Estimated by Fully Adjusted Models and Stratified by For-Profit Hospices vs Nonprofit/Government-Owned Hospices^a^

Characteristic	For-profit^a^		Nonprofit/government-owned^a^		*P* value for-profit vs nonprofit^b^
	Estimate (95% CI)	*P* value	Estimate (95% CI)	*P* value	
**Percentage of new monthly enrollees with ADRD**					
Pre-FTT/debility exclusion slope^c^	0.07 (0.06 to 0.08)	<.001	0.03 (0.02 to 0.04)	<.001	<.001
Change during FTT/debility exclusion notice	4.21 (3.70 to 4.72)	<.001	3.10 (2.60 to 3.61)	<.001	.002
Post-FTT/debility exclusion slope^c^	0.14 (0.08 to 0.19)	<.001	−0.01 (−0.06 to 0.04)	.74	<.001
Change during IMPACT Act passage	−1.86 (−2.84 to −0.88)	<.001	−0.59 (−1.49 to 0.31)	.20	.18
Change during IMPACT Act implementation	−1.88 (−2.87 to -0.89)	<.001	−2.13 (−3.05 to −1.21)	<.001	.75
Post–IMPACT Act slope^c^	0.13 (0.08 to 0.18)	<.001	0.07 (0.02 to 0.12)	.01	.06
Change during TTP implementation	0.07 (−0.41 to 0.55)	.77	0.36 (−0.12 to 0.83)	.14	.26
Post TTP-slope^c^	0.01 (0.00 to 0.02)	.14	0.01 (0.00 to 0.02)	.105	.64
**Percentage of monthly patient census with ADRD**					
Pre-FTT/debility exclusion slope^c^	0.15 (0.14 to 0.16)	<.001	0.08 (0.08 to 0.09)	<.001	<.001
Change during FTT/debility exclusion notice	3.15 (2.86 to 3.45)	<.001	2.79 (2.47 to 3.11)	<.001	.33
Post-FTT/debility exclusion slope^c^	0.2 (0.17 to 0.23)	<.001	−0.01 (−0.04 to 0.02)	.46	<.001
Change during IMPACT Act passage	−0.79 (−1.36 to −0.22)	.007	−0.53 (−1.13 to 0.07)	.08	.78
Change during IMPACT Act implementation	−1.31 (−1.88 to −0.73)	<.001	−1.80 (−2.42 to −1.19)	<.001	.59
Post–IMPACT Act slope^c^	0.02 (0.00 to 0.05)	.104	0.10 (0.07 to 0.13)	<.001	.02
Change during TTP implementation	−0.38 (−0.66 to −0.11)	.006	−0.23 (−0.55 to 0.08)	.14	.96
Post TTP-slope^c^	0.02 (0.01 to 0.02)	<.001	0.03 (0.02 to 0.04)	<.001	<.001
**Percentage of monthly care days provided to patients with ADRD**					
Pre-FTT/debility exclusion slope^c^	0.16 (0.16 to 0.17)	<.001	0.10 (0.10 to 0.11)	<.001	<.001
Change during FTT/debility exclusion notice	3.21 (2.89 to 3.54)	<.001	2.94 (2.57 to 3.30)	<.001	.72
Post-FTT/debility exclusion slope^c^	0.20 (0.17 to 0.23)	<.001	−0.01 (−0.05 to 0.03)	.55	<.001
Change during IMPACT Act passage	−0.50 (−1.12 to 0.12)	.113	−0.57 (−1.27 to 0.12)	.11	.89
Change during IMPACT Act implementation	−1.27 (-1.9 to −0.64)	<.001	−1.69 (−2.40 to −0.98)	<.001	.57
Post–IMPACT Act slope^c^	0.00 (−0.03 to 0.03)	.98	0.11 (0.07 to 0.15)	<.001	<.001
Change during TTP implementation	−0.38 (−0.67 to −0.08)	.01	−0.35 (−0.71 to 0.01)	.06	.37
Post TTP-slope^c^	0.01 (0.01 to 0.02)	<.001	0.04 (0.03 to 0.05)	<.001	<.001

Abbreviations: ADRD, Alzheimer disease and related dementias; FTT, failure-to-thrive and debility exclusion as a primary hospice diagnosis; IMPACT, Improving Medicare Post-Acute Care Transformation Act; TTP, 2-tier payment.

^a^ Adjusted for month indicators, (absorbed) hospice fixed effects and time-varying hospice-level covariates (hospice ownership, total registered nurses, total employees, new enrollee demographic distributions [age, sex, race and ethnicity], percentage of care days provided at routine home care and continuous home care levels, and percentage of care days provided in the community/home setting, nonskilled long-term care setting, and skilled nursing home/inpatient setting) and time-varying county-level covariates (population, percent population older than 65 years and older than 85 years, sex and race and ethnicity distributions, number of active physicians, rural-urban setting, percentage living below the poverty level, median household income).

^b^ For each policy indicator/slope, *P *values indicate significance of differences between for-profit vs nonprofit/government-owned hospices. They are *P *values associated with the interaction term between hospice ownership and each corresponding policy indicator/slope term in analyses on the full sample of all hospices, excluding absorbed hospice fixed effects, and fully adjusted for covariates identical to those used in the fully stratified analyses.

^c^ Slope coefficients are direct estimates of the slope during each specified period.

## Discussion

First, there was evidence of code-shifting behavior, with a steep increase in all outcomes (share of patients with ADRD among new enrollees, total patient census, and total care days) immediately following the FTT/debility exclusion notice,^16^ with particularly pronounced effects among high institutionalization hospices. This is consistent with the fact that Alzheimer disease was a commonly used contributing diagnoses in patients with a principal FTT/debility diagnosis in 2012.^16^ Additionally, because rates of increase in the share of patients with ADRD were similar during the months leading to vs the months following the notice to exclude FTT/debility, resulting changes in coding appeared to occur quickly and were established well before IMPACT Act passage. Thus, confounding by FTT/debility exclusion effects on the estimates of changes around later policies was likely minimal.

Second, IMPACT Act passage and implementation were associated with immediate reductions, followed by an increasing trend in all measures of the share of patients with ADRD. However, the descriptive analyses suggest a nonlinear trend, which is consistent with an immediate response to mitigate audit risk (ie, a “chilling” effect reducing and delaying patient enrollments^23,30,31,32^) followed by an increasing trend over time, likely reflecting successful adaptation by hospices to the new regulatory environment. Interestingly, high institutionalization hospices appeared to respond by reducing ADRD enrollment earlier (at the time of passage) than low institutionalization hospices (at the time of implementation).

Third, while there was negligible change in enrollments of patients with ADRD at the time of 2-tier payment implementation, there was a slight drop in both caseload outcomes. During the subsequent 4 years, all 3 measures rose at rates slower than those observed during the baseline period. This may reflect the effects of the new regulatory and financial pressures targeting long stays and/or a slowing in the rate of increase in demand among patients with ADRD for hospice care.

Fourth, there was evidence of stronger policy effects in for-profit hospices compared with nonprofit/government-owned hospices. Consistent with prior studies,^23,24,25,33,34,35^ for-profit hospices had persistently higher shares of patients with ADRD and exhibited faster growth of patients with ADRD during the pre-IMPACT period, during which long-stay patients generated larger profits. Moreover, for-profit hospices had slower growth in caseload outcomes during the post-IMPACT and post 2-tier payment periods, during which the residence of long-stay patients became more scrutinized and less profitable. These differences are important to consider given that most of the growth in the hospice industry during the analytical period was associated with new for-profit hospices.^36^

Overall, the study suggests that there was a decline in patients receiving hospice care with a primary or secondary diagnosis of ADRD associated with implementation of the IMPACT Act and 2-tier payment system, particularly at for-profit hospices. This may reflect reductions in enrollment of patients with ADRD who did not truly meet eligibility criteria (ie, reduced eligibility), as the policy intended. Alternatively, it may reflect unintended reduced access for hospice-appropriate patients with ADRD as hospices attempt to minimize risk of long stays, or some combination of these mechanisms. We expect that reduced hospice utilization for patients with ADRD, particularly among hospice-eligible patients, has been negatively associated with quality-of-life for patients and caregivers. In addition to limitations in cognition, communication, and ability to perform basic activities, patients with ADRD experience a complex combination of psychological (eg, depression, confusion, agitation, and behavioral changes) and physical symptoms (eg, pain, dyspnea, choking/coughing, and infections), posing uniquely burdensome demands on family caregivers.^1,2,3,4,5,6,7,9,37,38,39,40,41,42,43,44,45,46^ For these patients, hospice care, in prioritizing patient end-of-life comfort, goals, and patient-and-family holistic well-being, more readily facilitates quality of life than conventional care.^1,3,4,7,8,10,37,39,44,46,47,48^ However, this may not be the case if reductions in hospice utilization primarily reflect reduced eligibility of patients with an earlier stage of ADRD with lower symptom burdens.

We found evidence of reduced use of hospices among patients with ADRD with recent regulatory/payment changes to the hospice benefit. However, reductions were modest and potentially explainable by rational administrative behaviors, such as reducing enrollment of patients who were earlier in the disease trajectory. Further investigation is necessary to better understand how observed reductions in ADRD hospice utilization translate to patient and family experiences.

Many other questions also remain unanswered. First, it is unclear whether the study results are generalizable to other patient groups with relatively long hospice stays, including those with coronary heart failure and chronic obstructive pulmonary disease. Second, it is important to examine the consequences and mechanisms through which hospices have implemented change in response to the policies. Examination of length of stay and live discharges (which can be leveraged to reduce patient length of stay), as well as hospice profitability trends by patient mix, are of interest; summary statistics reported by the Medicare Payment Advisory Commission do not show declines in patient length of stay or generated profitability among hospices with longer lengths of stay,^49,50,51^ as the policies intended. Finally, it is important to consider and investigate alternative hospice policy solutions that can reduce, rather than increase, barriers to care and better facilitate a high-quality end of life for the growing ADRD population in a cost-effective manner. However, because we leveraged 100% Medicare claims data, generalizability to the US Medicare population is not a concern.

### Limitations

First, IMPACT Act implementation coincided with new hospice rules that excluded use of certain dementia codes (senile/presenile, vascular, and unspecified dementia) as principal diagnoses.^52^ However, the similarity of findings across primary analyses (using principal and secondary diagnoses codes) and sensitivity analyses (using additional diagnosis codes), suggests that code shifting did not greatly affect those identified as admitted with ADRD. Second, we used claims *ICD-9* and *ICD-10* codes to identify patients with ADRD in hospice, which do not necessarily capture the true underlying population of patients with ADRD. For example, the hospice population with ADRD *ICD-9* and *ICD-10* codes clearly changed with the notice of FTT/debility exclusion. Many patients with ADRD also have coexisting conditions (eg, cancer, heart disease)^53,54^ that may be less prone to regulatory scrutiny. Thus, changes in observed trends in patients receiving hospice care with an ADRD *ICD-9* or *ICD-10* code may partially reflect changes in documentation and coding practices of hospices concurrent with or in response to policies of interest, thereby concealing true changes in the use of hospice by patients with terminal ADRD. Third, given the short period between the implementation of the IMPACT Act and 2-tier payment system, it is unclear whether the 2-tier payment would have elicited greater response if enacted in isolation. Fourth, effects of the growing scrutiny around patients with long stays in hospice during the years before the IMPACT Act may be reflected in prepolicy trends, thus muting its effect. Fifth, although the IMPACT Act incorporated multiple policy changes, we assume the long-stay audits were associated with changes in patient mix. For example, the IMPACT Act also increased the frequency of hospice surveys. However, because surveys focus on hospice compliance around care quality, health/safety, and organizational environment, this mandate is unlikely to be strongly associated with the sensitivity of hospices around long hospice stays. Finally, the data did not allow us to observe concurrent trends in the proportion/number of Medicare-covered decedents with ADRD, precluding adjustment for trends in demand among patients with ADRD for hospice care or broader examination of coding changes.

## Conclusions

In this cross-sectional study of Medicare hospice claims data, we found evidence to suggest that recent Medicare policy changes targeting patients with long stays in hospice care were associated with lasting reductions in the share of patients receiving hospice care admitted with an ADRD code compared with expectations based on preimplementation trends. Future research should examine the mechanisms through which hospices enacted change and consequences for quality of care.

## Notes

Supplement. **eAppendix 1.** Detailed model specification **eAppendix 2.** Detailed summary of sensitivity analyses **eTable 1.** Number and percent of hospice episode exclusions **eTable 2. **Characteristics of hospice episodes before and after exclusions **eTable 3. **List of ICD-9 and ICD-10 codes used to identify ADRD **eTable 4. **Summary of time-varying hospice-level and county-level covariates **eTable 5. **Summary of hospice characteristics **eTable 6. **Differences in the trajectories of percent new monthly enrollees with ADRD, percent monthly patient census with ADRD, and percent care days provided to patients with ADRD coinciding with recent policy changes affecting the Medicare hospice benefit, estimated by fully adjusted models, using alternate ADRD identification and unique hospice episode identification strategies **eTable 7. **Differences in the trajectories of percent new monthly enrollees with ADRD, percent monthly patient census with ADRD, and percent care days provided to patients with ADRD coinciding with recent policy changes affecting the Medicare hospice benefit, estimated by fully adjusted modelsa using different subsamples of the data **eTable 8. **Differences in the trajectories of percent new monthly enrollees with ADRD, percent monthly patient census with ADRD, and percent care days provided to patients with ADRD coinciding with recent policy changes affecting the Medicare hospice benefit, estimated by fully adjusted models, within hospices with low baseline proportion of institutionalized patients, and hospice with high baseline proportion of institutionalized patients
eReferences
