# Supplementary material for: Evaluation of Federal Policy Changes to the Hospice Benefit and Use of Hospice for Persons With ADRD
Source: JAMA Health Forum. 2022 May 6;3(5):e220900. doi: 10.1001/jamahealthforum.2022.0900 (PMC9077487; doi:10.1001/jamahealthforum.2022.0900)
Supplement: Supplement. — eAppendix 1. Detailed model specification eAppendix 2. Detailed summary of sensitivity analyses eTable 1. Number and percent of hospice episode exclusions eTable 2. Characteristics of hospice episodes before and after exclusions eTable 3. List of ICD-9 and ICD-10 codes used to identify ADRD eTable 4. Summary of time-varying hospice-level and county-level covariates eTable 5. Summary of hospice characteristics eTable 6. Differences in the trajectories of percent new monthly enrollees with ADRD, percent monthly patient census with ADRD, and percent care days provided to patients with ADRD coinciding with recent policy changes affecting the Medicare hospice benefit, estimated by fully adjusted models, using alternate ADRD identification and unique hospice episode identification strategies eTable 7. Differences in the trajectories of percent new monthly enrollees with ADRD, percent monthly patient census with ADRD, and percent care days provided to patients with ADRD coinciding with recent policy changes affecting the Medicare hospice benefit, estimated by fully adjusted modelsa using different subsamples of the data eTable 8. Differences in the trajectories of percent new monthly enrollees with ADRD, percent monthly patient census with ADRD, and percent care days provided to patients with ADRD coinciding with recent policy changes affecting the Medicare hospice benefit, estimated by fully adjusted models, within hospices with low baseline proportion of institutionalized patients, and hospice with high baseline proportion of institutionalized patients eReferences [file jamahealthforum-e220900-s001.pdf]

Supplemental Online Content

Gianattasio KZ, Moghtaderi A, Lupu D, Prather C, Power MC. Evaluation of federal policy changes to the hospice benefit and use of hospice for persons with ADRD. *JAMA Health Forum*. 2022;3(5):e220900.
doi:10.1001/jamahealthforum.2022.0900

eAppendix 1. Detailed model specification
eAppendix 2. Detailed summary of sensitivity analyses
eTable 1. Number and percent of hospice episode exclusions
eTable 2. Characteristics of hospice episodes before and after exclusions
eTable 3. List of ICD-9 and ICD-10 codes used to identify ADRD
eTable 4. Summary of time-varying hospice-level and county-level covariates
eTable 5. Summary of hospice characteristics
eTable 6. Differences in the trajectories of percent new monthly enrollees with ADRD, percent monthly patient census with ADRD, and percent care days provided to patients with ADRD coinciding with recent policy changes affecting the Medicare hospice benefit, estimated by fully adjusted models, using alternate ADRD identification and unique hospice episode identification strategies
eTable 7. Differences in the trajectories of percent new monthly enrollees with ADRD, percent monthly patient census with ADRD, and percent care days provided to patients with ADRD coinciding with recent policy changes affecting the Medicare hospice benefit, estimated by fully adjusted modelsa using different subsamples of the data
eTable 8. Differences in the trajectories of percent new monthly enrollees with ADRD, percent monthly patient census with ADRD, and percent care days provided to patients with ADRD coinciding with recent policy changes affecting the Medicare hospice benefit, estimated by fully adjusted models, within hospices with low baseline proportion of institutionalized patients, and hospice with high baseline proportion of institutionalized patients
eReferences

This supplemental material has been provided by the authors to give readers additional information about their work.

© 2022 Gianattasio KZ et al. *JAMA Health Forum.*

eAppendix 1. Detailed model specification

We considered the trends in each of our three outcomes over time, in relation to the FTT/debility exclusion notice published in May 2013 (to account for code-shifting towards increased use of ADRD codes), the passage/implementation of the IMPACT ACT in September/October 2014, and implementation of the two-tier payment system in January 2016.
We modelled trends in each outcome as parametrized below:

$$Y_{ict} = \beta_0 + \beta_1 t\_pp_t + \beta_2 FTT_t + \beta_3 t\_FTT_t + \beta_4 IMPpass_t + \beta_5 IMPimp_t + \beta_6 t\_IMPimp_t + \beta_7 newPay_t + \beta_8 t\_newPay_t + \gamma + \delta(H_{it}) + \vartheta(C_{ct}) + \alpha_i + \varepsilon_{ict} \quad [1]$$

where *i* indexes hospice, *c* indexes county, *t* indexes time in months, and Y is the outcome of interest. We used linear splines to model the linear trend in our outcome over the four distinct time periods demarcated by FTT/debility exclusion notice, IMPACT Act passage/implementation, and two-tier payment implementation: *t_pp* for the pre-FTT/debility exclusion notice time period, *t_FTT* for the post-FTT/debility exclusion notice period, *t_IMPimp* for the post-IMPACT ACT period, and *t_newPay* for the post-two-tier payment system implementation period. We also included indicator terms to allow for and estimate immediate changes in our outcomes at the time of the FTT/debility exclusion notice, passage of the IMPACT Act, implementation of the IMPACT Act, and implementation of the two-tier payment system: *FTT*, *IMPpass*, *IMPimp*, and *newPay*. We adjusted for several covariates: γ represents month fixed-effects to account for seasonality, and *H* and *C* represent time-varying hospice- and county-level characteristics (**Appendix Table 4**). Finally, to estimate within-hospice variations across time, we included hospice fixed-effects (α).[31]

© 2022 Gianattasio KZ et al. *JAMA Health Forum.*

eAppendix 2. Detailed summary of sensitivity analyses

First, because ADRD patients often have other contributing comorbidities, we repeated the analyses by classifying as ADRD patients all those with ADRD coded in (i) the principal, second, or third diagnoses codes, and (ii) the principal, second, third, or fourth diagnoses codes. Second, for purposes of determining payment rate under the new two-tier payment system, re-enrollments within 60 days after a discharge are considered as a continuation of the prior episode of care. Thus, we used an alternative strategy to identify new enrollments, which requires any re-enrollment following a prior discharge or break in claims to occur at least 60 days apart, and repeated our analysis of trends in percent new enrollees with ADRD. Third, we repeated our analyses for all outcomes excluding data from government-owned hospices (4.4% of all hospices), which may operate fundamentally differently from privately-owned non-profit hospices. Similarly, as beneficiaries covered by Medicare Advantage (MA) may experience different terminal care and hospice referral practices to those covered by traditional fee for service (FFS) Medicare, we repeated our analyses for all outcomes excluding beneficiaries with any MA coverage (accounting for 30.9% of all episodes). Fifth, to eliminate the influence of hospices that newly open or shut down during the analytical period (July 2008 – December 2019), we repeated our analyses limiting to hospices providing care throughout the observation period. Sixth, given that 2008-2009 claims only contain date of service quarter indicators rather than specific dates (which we converted to month indicators used in our primary analyses by random number generation), we repeated our analyses excluding data for hospice episodes that began prior to 2010. Finally, because scrutiny has historically been especially pronounced around long-stay patients that receive hospice care in institutionalized settings (e.g. long-term care facilities, skilled nursing facilities), we explored potential differences in policy effects between hospices with low vs. high baseline proportion of care provided to patients in institutionalized settings. Specifically, we defined the baseline period as July 2008 – April 2013, and excluded hospices that entered the market within two years of the end of this period (i.e. after April 2011). We then identified hospices with low proportion of institutionalized patients as those that had lower than median percentage (38%) of care provided to patients in institutionalized settings in at least 90% of months during the baseline period. Conversely, hospices with high baseline proportion institutionalized care were those that provided higher than the median percentage (38%) of care to institutionalized patients in at least 90% of months during the baseline period.

© 2022 Gianattasio KZ et al. *JAMA Health Forum.*

eTable 1. Number and percent of hospice episode exclusions

Year	Total	Exclusions, N(%)						Remaining in sample, N(%)
		Missing hospice-level covariates	Missing county-level covariates	Missing demographics or age < 65	Claim date discrepancies	Claim date and death date discrepancies	Claims with out-of-range claim dates or claim days	
Overall	13,812,688	1,453,842 (10.5%)	132,411 (1.0%)	801,101 (5.8%)	34,595 (0.3%)	59,246 (0.4%)	206,571 (1.5%)	11,124,922 (80.5%)
2008	468,799	35,602 (7.6%)	7,096 (1.5%)	29,597 (6.3%)	172 (0%)	2,005 (0.4%)	11,179 (2.4%)	383,148 (81.7%)
2009	984,837	79,964 (8.1%)	10,886 (1.1%)	58,455 (5.9%)	411 (0%)	4,536 (0.5%)	28,484 (2.9%)	802,101 (81.4%)
2010	1,040,677	94,714 (9.1%)	12,804 (1.2%)	60,822 (5.8%)	1,748 (0.2%)	11,353 (1.1%)	19,330 (1.9%)	839,906 (80.7%)
2011	1,092,856	109,024 (10%)	12,957 (1.2%)	64,915 (5.9%)	1,736 (0.2%)	10,969 (1%)	19,935 (1.8%)	873,320 (79.9%)
2012	1,137,921	119,388 (10.5%)	13,833 (1.2%)	69,498 (6.1%)	1,837 (0.2%)	6,855 (0.6%)	16,875 (1.5%)	909,635 (79.9%)
2013	1,177,293	126,300 (10.7%)	13,938 (1.2%)	71,397 (6.1%)	1,868 (0.2%)	3,450 (0.3%)	15,595 (1.3%)	944,745 (80.2%)
2014	1,189,571	134,157 (11.3%)	14,264 (1.2%)	71,056 (6.0%)	2,506 (0.2%)	3,230 (0.3%)	14,776 (1.2%)	949,582 (79.8%)
2015	1,251,076	150,616 (12%)	14,283 (1.1%)	72,381 (5.8%)	3,034 (0.2%)	3,342 (0.3%)	15,416 (1.2%)	992,004 (79.3%)
2016	1,288,323	144,446 (11.2%)	8,178 (0.6%)	73,298 (5.7%)	6,465 (0.5%)	5,334 (0.4%)	16,617 (1.3%)	1,033,985 (80.3%)
2017	1,345,534	137,588 (10.2%)	7,611 (0.6%)	75,933 (5.6%)	3,477 (0.3%)	4,453 (0.3%)	16,773 (1.2%)	1,099,699 (81.7%)
2018	1,396,326	154,200 (11%)	8,092 (0.6%)	76,508 (5.5%)	5,923 (0.4%)	2,001 (0.1%)	17,044 (1.2%)	1,132,558 (81.1%)
2019	1,439,475	167,843 (11.7%)	8,469 (0.6%)	77,241 (5.4%)	5,418 (0.4%)	1,718 (0.1%)	14,547 (1.0%)	1,164,239 (80.9%)

© 2022 Gianattasio KZ et al. *JAMA Health Forum.*

eTable 2. Characteristics of hospice episodes before and after exclusions [a]

	**All episodes**	**Exclude episodes missing hospice-covariates**	**Exclude missing county covariates**	**Exclude age < 65 or missing demographic**	**Exclude claim date and death date discrepancies**	**Exclude out-of-range claim dates or claim days (remaining claims)**
N	13,812,688	12,358,846	12,226,435	11,425,334	11,331,493	11,124,922
Patient age at enrollment, mean (SD)	81 (10.5)	81 (10.5)	81 (10.5)	82.7 (8.5)	82.7 (8.5)	82.7 (8.5)
Male patients, %	42.50%	42.40%	42.40%	41.60%	41.60%	41.70%
Patient race/ethnicity						
White, %	86.70%	86.50%	86.80%	87.50%	87.50%	87.50%
Black, %	8.50%	8.50%	8.50%	8.00%	8.00%	7.90%
Hispanic, %	2.00%	2.10%	1.80%	1.80%	1.80%	1.80%
Other, %	2.80%	2.90%	2.80%	2.80%	2.80%	2.80%
Patient had any MA coverage, %	30.90%	30.90%	30.60%	30.90%	30.90%	30.90%
Patient with ADRD, %	20.50%	20.50%	20.40%	21.50%	21.50%	21.40%
Region						
Northeast, %	15.60%	15.60%	15.70%	15.90%	15.90%	16.00%
West, %	20.10%	20.00%	20.10%	20.40%	20.40%	20.30%
South, %	40.60%	40.70%	40.50%	40.00%	40.00%	39.80%
Midwest, %	23.70%	23.60%	23.70%	23.80%	23.80%	23.90%
Rurality						
Large metro, %	88.50%	88.50%	88.40%	88.60%	88.60%	88.60%
Urban, adjacent to metro, %	6.90%	6.90%	6.90%	6.90%	6.90%	6.90%
Urban, nonadjacent to metro, %	4.20%	4.20%	4.20%	4.10%	4.10%	4.10%
Rural, %	0.40%	0.40%	0.40%	0.40%	0.40%	0.40%
Hospice ownership						
For-profit, %	49.80%	49.70%	49.70%	49.60%	49.60%	49.30%
Non-profit, %	48.40%	48.40%	48.40%	48.50%	48.50%	48.80%
Government-owned, %	1.90%	1.90%	1.90%	1.90%	1.90%	1.90%
Freestanding, %	79.10%	79.20%	79.10%	79.10%	79.10%	79.00%

Abbreviations: ADRD = Alzheimer's Disease and Related Dementias; MA = Medicare Advantage; SD = standard deviation

[a] Exclusions are cumulative across columns.

© 2022 Gianattasio KZ et al. *JAMA Health Forum.*

eTable 3. List of ICD-9 and ICD-10 codes used to identify ADRD

This list was constructed by expanding on a previously validated set of ICD-9 indicating ADRD prior to the ICD-9/ICD-10 shift,[1] and identifying their ICD-10 equivalents for use in claims after September 2015

ICD-9		ICD-10	
**Code**	**Description**	**Code**	
331.0	Alzheimer's disease	G30.9	Alzheimer's disease, unspecified
		G30.0	Alzheimer's disease with early onset
		G30.1	Alzheimer's diseas3 with late onset
		G30.8	Other Alzheimer's disease
331.1	Frontotemporal dementia	G31.0	
331.11	Pick's disease	G31.01	Pick's disease
331.19	Other frontotemporal dementia	G31.09	Other frontotemporal dementia
331.2	Senile degeneration of the brain	G31.1	Senile degeneration of brain, not elsewhere classified
331.7	Cerebral degeneration in diseases classified elsewhere	G94	Other disorders of brain in diseases classified elsewhere
331.82	Dementia with Lewy bodies	G31.83	Dementia with Lewy bodies
290.0	Senile dementia, uncomplicated	F03 .90	Unspecified dementia, without behavioral disturbance
290.1	Presenile dementia (brain syndrome with presenile dementia)		
290.10	Presenile dementia, uncomplicated		
290.11	Presenile dementia, with delirium		
290.12	Presenile dementia, with delusional features		
290.13	Presenile dementia, with depressive features		
290.20	Senile dementia, with delusional features		
290.21	Senile dementia, with depressive features		
290.3	Senile dementia, with delirium		
290.40	Arteriosclerotic dementia, uncomplicated	F01.50	Vascular dementia, without behavioral disturbance
290.41	Arteriosclerotic dementia, with delirium	F01.51	Vascular dementia, with behavioral disturbance
290.42	Arteriosclerotic dementia, with delusional features		
290.43	Arteriosclerotic dementia, with depressive features		
290.8	Other specified senile psychotic conditions	F03.90	Unspecified dementia without behavioral disturbance
290.9	Unspecified senile psychotic condition		
294.0	Amnestic syndrome (Korsakoff's psychosis or syndrome, nonalcoholic)	F04	Amnestic disorder due to known physiological condition
294.1	Dementia in conditions classified elsewhere		

© 2022 Gianattasio KZ et al. *JAMA Health Forum.*

294.10	Dementia in conditions classified elsewhere, without behavioral disturbance	F02.80	Dementia in other diseases classified elsewhere, without behavioral disturbance
294.11	Dementia in conditions classified elsewhere, with behavioral disturbance	F02.81	Dementia in other diseases classified elsewhere, with behavioral disturbance
294.2	Dementia, unspecified		
294.20	Dementia, unspecified, without behavioral disturbance	F03.90	Unspecified dementia, without behavioral disturbance
294.21	Dementia, unspecified, with behavioral disturbance	F03.91	Unspecified dementia, with behavioral disturbance
294.8	Other persistent mental disorders due to conditions classified elsewhere	F06.0	Psychotic disorder with hallucinations due to known physiological condition
		F06.8	Other specified mental disorders due to known physiological condition
797	Senility without mention of psychosis	R41.81	Age-related cognitive decline

© 2022 Gianattasio KZ et al. *JAMA Health Forum.*

eTable 4. Summary of time-varying hospice-level and county-level covariates

**Time-varying hospice-level covariates**	
Hospice profit status	Binary variable: 0=non-profit/government-owned; 1=for-profit
Hospice affiliation	Binary variable: 0=affiliated with another entity; 1=freestanding
Number of full-time equivalent registered nurses employed by the hospice each year	Continuous variable: logarithm
Number of full-time equivalent employees employed by the hospice each year	Continuous variable: logarithm
Average age of patients newly enrolled each month	Continuous variable
Percent of newly enrolled patients each month who are white	Continuous variable
Percent of newly enrolled patients each month who are Black	Continuous variable
Percent of newly enrolled patients each month who are Hispanic	Continuous variable
Percent of newly enrolled patients each month who are male	Continuous variable
Percent of care days provided each month at the routine home care level	Continuous variable
Percent of care days provided each month at the continuous home care level	Continuous variable
Percent of care days provided to patients in a home setting	Continuous variable
Percent of care days provided to patients in a non-skilled long-term care setting	Continuous variable
Percent of care days provided to patients in a skilled nursing facility or inpatient setting	Continuous variable
**Time-Varying county-level covariates**	
Total population	Continuous variable: logarithm
Percent of population aged 65+	Continuous variable
Percent of population aged 85+	Continuous variable
Percent of population male	Continuous variable
Percent of population of white race	Continuous variable
Percent of population of Black race	Continuous variable
Percent of population of Hispanic ethnicity	Continuous variable
Total number of non-federal active physicians (MDs)	Continuous variable: logarithm
Percent of population below poverty line	Continuous variable
Household median income	Continuous variable: logarithm
Rural-urban continuum code	Categorical variable:<br>1: Metropolitan, pop >= 1 mn<br>2: Metropolitan, 250k <= pop < 1 mn<br>3: Metropolitan, pop < 250k<br>4: Urban, pop >= 20k, adjacent to metropolitan<br>5: Urban, pop >= 20k, NOT adjacent to metropolitan<br>6: Urban, 2.5k <= pop < 20k, adjacent to metropolitan<br>7: Urban, 2.5k <= pop < 20k, NOT adjacent to metropolitan<br>8: Rural OR population < 2.5k, adjacent to metropolitan<br>9: Rural OR population < 2.5k, NOT adjacent to metropolitan

© 2022 Gianattasio KZ et al. *JAMA Health Forum.*

eTable 5. Summary of hospice characteristics

	2008	2009	2010	2011	2012	2013	2014	2015	2016	2017	2018	2019
N	2,984	3,027	3,097	3,161	3,272	3,420	3,585	3,655	3,824	3,992	4,127	4,303
Monthly patient census	62.5 (103.1)	72.8 (140.2)	78.5 (154.6)	80.5 (159.8)	83.3 (163.4)	82 (159.5)	78.4 (159.5)	79.7 (169.9)	80.8 (182.2)	82.7 (188.9)	84.4 (200.5)	85.7 (213.3)
Monthly number of care days provided	1303 (2161.4)	1578.4 (3138.3)	1718.5 (3511.7)	1777.1 (3656.2)	1858.5 (3761.1)	1815 (3646.3)	1732.5 (3653.7)	1760.6 (3926.4)	1799.6 (4280)	1839.4 (4439)	1893.6 (4753.9)	1944.3 (5090.7)
RN FTE, mean (SD)	8.8 (17.5)	9.3 (19.3)	9.7 (19.9)	10 (19.9)	10.2 (20.6)	10.4 (20.5)	10.3 (20.1)	10.6 (20.4)	10.5 (20.9)	10.4 (20.5)	10.3 (21.4)	10.1 (20.2)
MD FTE, mean (SD)	1.1 (2.6)	1.1 (2.6)	1.2 (2.6)	1.2 (2.7)	1.5 (11.2)	1.5 (11.3)	1.4 (11)	1.4 (10.8)	1.4 (10.6)	1.2 (2.5)	1.1 (2.1)	1.1 (1.8)
Total employees, mean (SD)	28.3 (61.3)	29.7 (63.7)	30.9 (65.6)	33.3 (90.9)	34.1 (92.7)	34.3 (89.5)	34.2 (89)	33.4 (66.4)	33.3 (69.3)	32.4 (66.9)	31.7 (66.7)	30.3 (62.2)
Level of care												
% RHC, mean (SD)	98.2 (3.6)	98.2 (3)	97.7 (3.9)	97.7 (3.8)	98.2 (4)	98.5 (2.9)	98.5 (3)	98.6 (3.1)	98.6 (3.1)	98.7 (3.7)	98.8 (3.1)	98.9 (3.1)
% CHC, mean (SD)	0.1 (0.5)	0.2 (1.1)	0.2 (0.7)	0.2 (0.9)	0.2 (1.1)	0.2 (0.9)	0.2 (1)	0.2 (0.9)	0.2 (1.2)	0.2 (2.1)	0.2 (1.2)	0.2 (1.3)
% inpatient, mean (SD)	1.3 (3.4)	1.3 (2.7)	1.3 (2.7)	1.3 (2.8)	1.2 (3.7)	1.1 (2.6)	1 (2.7)	1 (2.8)	0.9 (2.7)	0.8 (2.9)	0.7 (2.7)	0.7 (2.6)
% respite, mean (SD)	0.2 (0.4)	0.2 (0.3)	0.2 (0.4)	0.3 (0.4)	0.3 (0.4)	0.3 (0.5)	0.3 (0.4)	0.3 (0.4)	0.3 (0.5)	0.3 (0.5)	0.3 (0.9)	0.3 (0.5)
Place of care												
% home, mean (SD)	61.6 (26.5)	62.1 (26.5)	61.4 (26.6)	61.8 (26)	62.2 (26.1)	62.3 (26.1)	62.7 (25.8)	62.4 (25.8)	62.3 (26.3)	62.1 (26.1)	62.2 (26.3)	62.4 (26.7)
% non-skilled LTC, mean (SD)	24.8 (24.8)	25 (24.7)	26.5 (24.9)	27 (25.1)	27.4 (25.3)	27.8 (25.2)	27.4 (24.8)	27.8 (24.9)	28 (25.1)	28.9 (25.1)	29.3 (25.4)	29.4 (25.6)
% SNF or inpatient, mean (SD)	12.8 (19.4)	12.3 (18.6)	10.8 (16.2)	9.5 (15.5)	9.3 (15.2)	9 (14.5)	8.8 (13.8)	8.6 (14.1)	8.6 (14.4)	7.9 (13.7)	7.6 (13.3)	7.4 (12.9)
Region												
Northeast, %	13.30%	13.40%	13.30%	13.30%	13.00%	12.40%	11.70%	11.30%	10.70%	10.20%	9.70%	9.10%
West, %	18.80%	19.00%	20.00%	20.80%	21%	23.20%	25.10%	27.00%	29.10%	31.40%	33%	34.60%
South, %	42.70%	42.90%	42.60%	42.30%	42.10%	41.60%	41.00%	39.60%	38.50%	37.30%	36.90%	36.20%
Midwest, %	25.20%	25%	24.10%	23.70%	23.60%	22.90%	22%	22.10%	21.70%	21.10%	20.60%	20.10%
Rurality												
Large metro, %	69%	69.60%	71.20%	72.10%	73.00%	76.20%	77.40%	78.40%	79.60%	80.60%	81.10%	82.20%
Urban, metro adj., %	15.70%	15.70%	15%	14.60%	14.20%	12.60%	12%	11.40%	10.90%	11%	10.20%	9%
Urban, non-metro adj., %	12.20%	12.20%	11.40%	11.10%	10.80%	9.70%	9.40%	9%	8.30%	8%	8%	8%

© 2022 Gianattasio KZ et al. *JAMA Health Forum.*

Rural, %	2.80%	2.50%	2.40%	2.20%	2.00%	1.50%	1.50%	1.30%	1.20%	1.10%	1%	0.90%
Hospice ownership												
For-profit, %	54.80%	56.20%	57.80%	59.80%	62%	64.60%	67.00%	68.80%	70.00%	71.00%	73%	74.60%
Non-profit, %	39.10%	38.10%	36.60%	34.90%	33.00%	31%	28.60%	27.10%	26.10%	25.50%	23.80%	22.40%
Government, %	6.10%	5.70%	5.60%	5.30%	5.00%	4.70%	4.40%	4.10%	3.90%	3.50%	3.30%	3.00%
Freestanding, %	64.60%	66.60%	68.60%	70.40%	72.60%	74.90%	77.10%	79.40%	80.70%	82.60%	83.50%	84.50%

Abbreviations: CHC = continuous home care; FTT = failure-to-thrive/debility; LTC = long-term care facility; MD = medical doctor; RHC = routine home care; RN = registered nurse; SD = standard deviation; SNF = skilled nursing home

© 2022 Gianattasio KZ et al. *JAMA Health Forum.*

eTable 6. Differences in the trajectories of percent new monthly enrollees with ADRD, percent monthly patient census with ADRD, and percent care days provided to patients with ADRD coinciding with recent policy changes affecting the Medicare hospice benefit, estimated by fully adjusted models,[a] using alternate ADRD identification and unique hospice episode identification strategies

	**ADRD defined using principal and first two contributing diagnoses**		**ADRD defined using principal and first three contributing diagnoses**		**Alternate hospice episode identification strategy**	
	**Estimate (95% CI)**	**p-value**	**Estimate (95% CI)**	**p-value**	**Estimate (95% CI)**	**p-value**
Percent new monthly enrollees with ADRD						
Pre-FTT/debility exclusion slope [b]	0.06 (0.05, 0.07)	<.001	0.06 (0.06, 0.07)	<.001	0.05 (0.04, 0.06)	<.001
Change during FTT/debility exclusion notice	4.02 (3.64, 4.41)	<.001	4.22 (3.83, 4.61)	<.001	3.82 (3.44, 4.20)	<.001
Post-FTT/debility exclusion slope [b]	0.09 (0.05, 0.13)	<.001	0.09 (0.05, 0.13)	<.001	0.09 (0.05, 0.12)	<.001
Change during IMPACT passage	-1.28 (-2, -0.56)	<.001	-1.22 (-1.96, -0.49)	0.001	-1.37 (-2.10, -0.65)	<.001
Change during IMPACT implementation	-2.14 (-2.88, -1.4)	<.001	-2.33 (-3.09, -1.58)	<.001	-2.05 (-2.79, -1.31)	<.001
Post-IMPACT slope [b]	0.18 (0.14, 0.22)	<.001	0.25 (0.21, 0.29)	<.001	0.11 (0.07, 0.15)	<.001
Change during TTP implementation	0.38 (0.01, 0.75)	0.046	0.63 (0.26, 1.01)	0.001	0.21 (-0.15, 0.58)	0.248
Post TTP-slope [b]	0.01 (0, 0.02)	0.014	0.01 (0, 0.02)	0.003	0.01 (0, 0.02)	0.004
Percent monthly patient census with ADRD						
Pre-FTT/debility exclusion slope [b]	0.13 (0.13, 0.13)	<.001	0.14 (0.14, 0.14)	<.001	NA	
Change during FTT/debility exclusion notice	3.19 (2.96, 3.42)	<.001	3.30 (3.06, 3.53)	<.001		
Post-FTT/debility exclusion slope [b]	0.14 (0.12, 0.16)	<.001	0.15 (0.13, 0.18)	<.001		
Change during IMPACT passage	-0.63 (-1.07, -0.19)	0.005	-0.61 (-1.05, -0.16)	0.008		
Change during IMPACT implementation	-1.54 (-1.99, -1.09)	<.001	-1.62 (-2.08, -1.16)	<.001		
Post-IMPACT slope [b]	0.12 (0.10, 0.14)	<.001	0.19 (0.16, 0.21)	<.001		
Change during TTP implementation	-0.15 (-0.37, 0.07)	0.184	0.03 (-0.19, 0.25)	0.81		
Post TTP-slope [b]	0.02 (0.02, 0.03)	<.001	0.03 (0.02, 0.03)	<.001		
Percent monthly care days provided to patients with ADRD						
Pre-FTT/debility exclusion slope [b]	0.15 (0.14, 0.15)	<.001	0.16 (0.15, 0.16)	<.001	NA	
Change during FTT/debility exclusion notice	3.25 (3.00, 3.51)	<.001	3.36 (3.10, 3.61)	<.001		
Post-FTT/debility exclusion slope [b]	0.15 (0.12, 0.17)	<.001	0.16 (0.13, 0.19)	<.001		
Change during IMPACT passage	-0.47 (-0.96, 0.02)	0.058	-0.43 (-0.93, 0.06)	0.084		
Change during IMPACT implementation	-1.47 (-1.96, -0.97)	<.001	-1.53 (-2.03, -1.02)	<.001		
Post-IMPACT slope [b]	0.11 (0.09, 0.14)	<.001	0.18 (0.15, 0.20)	<.001		
Change during TTP implementation	-0.21 (-0.45, 0.03)	0.09	-0.07 (-0.31, 0.17)	0.574		
Post TTP-slope [b]	0.02 (0.02, 0.03)	<.001	0.03 (0.02, 0.03)	<.001		

Abbreviations: ADRD = Alzheimer's Disease and Related Dementias; FTT = failure-to-thrive and debility exclusion as a primary hospice diagnosis; NA = not applicable; TTP = two-tier pay

[a] Adjusted for month indicators, (absorbed) hospice fixed effects + time-varying hospice-level covariates (hospice ownership, total registered nurses, total employees, new enrollee demographic distributions (age, gender, race/ethnicity), percent care days provided at routine home care and continuous home care levels, and percent care days provided in the community/home setting, nonskilled long-term care setting, and skilled nursing home/inpatient setting) + time-varying county-level covariates (population, percent population over age 65 and over age 85, gender and race/ethnicity distribution, number of active physicians, rural-urban setting, percent below poverty level, median household income).

© 2022 Gianattasio KZ et al. *JAMA Health Forum.*

[b] Slope coefficients are direct estimates of the slope during each specified time-period

© 2022 Gianattasio KZ et al. *JAMA Health Forum.*

eTable 7. Differences in the trajectories of percent new monthly enrollees with ADRD, percent monthly patient census with ADRD, and percent care days provided to patients with ADRD coinciding with recent policy changes affecting the Medicare hospice benefit, estimated by fully adjusted models[a] using different subsamples of the data

	Excluding government-owned hospices		Excluding MA enrollees		Subset of hospices in operation between July 2008 and Dec 2019		Limited to data from 2010 and later	
	Estimate (95% CI)	p-value	Estimate (95% CI)	p-value	Estimate (95% CI)	p-value	Estimate (95% CI)	p-value
Percent new monthly enrollees with ADRD								
Pre-FTT/debility exclusion slope [b]	0.05 (0.04, 0.06)	<.001	0.06 (0.05, 0.06)	<.001	0.05 (0.04, 0.05)	<.001	0.06 (0.05, 0.07)	<.001
Change during FTT/debility exclusion notice	3.90 (3.52, 4.28)	<.001	3.93 (3.51, 4.36)	<.001	3.94 (3.56, 4.32)	<.001	3.68 (3.29, 4.06)	<.001
Post-FTT/debility exclusion slope [b]	0.09 (0.06, 0.13)	<.001	0.08 (0.03, 0.12)	<.001	0.07 (0.03, 0.11)	<.001	0.09 (0.06, 0.13)	<.001
Change during IMPACT passage	-1.5 (-2.22, -0.77)	<.001	-1.23 (-2.05, -0.41)	0.003	-1.80 (-2.49, -1.11)	<.001	-1.44 (-2.16, -0.73)	<.001
Change during IMPACT implementation	-1.97 (-2.71, -1.23)	<.001	-2.17 (-3.01, -1.34)	<.001	-1.57 (-2.27, -0.86)	<.001	-1.96 (-2.68, -1.23)	<.001
Post-IMPACT slope [b]	0.12 (0.08, 0.15)	<.001	0.10 (0.06, 0.15)	<.001	0.11 (0.07, 0.14)	<.001	0.11 (0.08, 0.15)	<.001
Change during TTP implementation	0.14 (-0.22, 0.51)	0.447	0.22 (-0.19, 0.64)	0.292	0.48 (0.12, 0.84)	0.009	0.18 (-0.18, 0.54)	0.334
Post TTP-slope [b]	0.01 (0.01, 0.02)	<.001	0.01 (0, 0.02)	0.003	0.02 (0.01, 0.03)	<.001	0.01 (0.01, 0.02)	<.001
Percent monthly patient census with ADRD								
Pre-FTT/debility exclusion slope [b]	0.12 (0.12, 0.13)	<.001	0.12 (0.12, 0.13)	<.001	0.12 (0.11, 0.12)	<.001	0.13 (0.12, 0.14)	<.001
Change during FTT/debility exclusion notice	3.12 (2.89, 3.34)	<.001	2.97 (2.72, 3.23)	<.001	3.01 (2.78, 3.24)	<.001	2.87 (2.64, 3.1)	<.001
Post-FTT/debility exclusion slope [b]	0.13 (0.11, 0.16)	<.001	0.13 (0.1, 0.16)	<.001	0.09 (0.07, 0.12)	<.001	0.13 (0.11, 0.15)	<.001
Change during IMPACT passage	-0.69 (-1.13, -0.25)	0.002	-0.42 (-0.92, 0.08)	0.102	-0.8 (-1.24, -0.36)	<.001	-0.73 (-1.16, -0.3)	<.001
Change during IMPACT implementation	-1.49 (-1.94, -1.05)	<.001	-1.49 (-2.00, -0.97)	<.001	-1.57 (-2.02, -1.12)	<.001	-1.44 (-1.87, -1)	<.001
Post-IMPACT slope [b]	0.05 (0.03, 0.07)	<.001	0.03 (0.01, 0.06)	0.019	0.07 (0.05, 0.1)	<.001	0.05 (0.03, 0.07)	<.001
Change during TTP implementation	-0.29 (-0.51, -0.08)	0.007	-0.34 (-0.59, -0.09)	0.008	-0.13 (-0.36, 0.09)	0.242	-0.34 (-0.54, -0.13)	0.002
Post TTP-slope [b]	0.03 (0.02, 0.03)	<.001	0.02 (0.02, 0.03)	<.001	0.02 (0.02, 0.03)	<.001	0.03 (0.02, 0.03)	<.001
Percent monthly care days provided to patients with ADRD								
Pre-FTT/debility exclusion slope [b]	0.14 (0.13, 0.14)	<.001	0.14 (0.13, 0.14)	<.001	0.13 (0.13, 0.14)	<.001	0.15 (0.14, 0.16)	<.001
Change during FTT/debility exclusion notice	3.22 (2.97, 3.47)	<.001	3.00 (2.72, 3.28)	<.001	3.14 (2.88, 3.4)	<.001	2.89 (2.63, 3.14)	<.001
Post-FTT/debility exclusion slope [b]	0.13 (0.11, 0.16)	<.001	0.14 (0.11, 0.17)	<.001	0.09 (0.06, 0.12)	<.001	0.13 (0.11, 0.16)	<.001
Change during IMPACT passage	-0.48 (-0.96, 0)	0.05	-0.27 (-0.82, 0.29)	0.349	-0.62 (-1.12, -0.12)	0.015	-0.55 (-1.02, -0.08)	0.023
Change during IMPACT implementation	-1.43 (-1.92, -0.94)	<.001	-1.39 (-1.96, -0.82)	<.001	-1.55 (-2.06, -1.04)	<.001	-1.38 (-1.86, -0.9)	<.001
Post-IMPACT slope [b]	0.03 (0.01, 0.06)	0.007	0.02 (-0.01, 0.05)	0.274	0.07 (0.05, 0.1)	<.001	0.04 (0.02, 0.07)	<.001
Change during TTP implementation	-0.30 (-0.53, -0.07)	0.012	-0.37 (-0.65, -0.1)	0.008	-0.23 (-0.47, 0.02)	0.074	-0.38 (-0.61, -0.15)	0.001
Post TTP-slope [b]	0.02 (0.02, 0.03)	<.001	0.02 (0.02, 0.03)	<.001	0.02 (0.02, 0.03)	<.001	0.03 (0.02, 0.03)	<.001

Abbreviations: ADRD = Alzheimer's Disease and Related Dementias; FTT = failure-to-thrive and debility exclusion as a primary hospice diagnosis; TTP = two-tier pay

[a] Adjusted for month indicators, (absorbed) hospice fixed effects + time-varying hospice-level covariates (hospice ownership, total registered nurses, total employees, new enrollee demographic distributions (age, gender, race/ethnicity), percent care days provided at routine home care and continuous home care levels, and percent care days provided in the community/home setting, nonskilled long-term care setting, and skilled nursing home/inpatient setting) + time-varying county-level covariates (population, percent population over age 65 and over age 85, gender and race/ethnicity distribution, number of active physicians, rural-urban setting, percent below poverty level, median household income).

[b] Slope coefficients are direct estimates of the slope during each specified time-period

© 2022 Gianattasio KZ et al. *JAMA Health Forum.*

eTable 8. Differences in the trajectories of percent new monthly enrollees with ADRD, percent monthly patient census with ADRD, and percent care days provided to patients with ADRD coinciding with recent policy changes affecting the Medicare hospice benefit, estimated by fully adjusted models,[a] within hospices with low baseline proportion of institutionalized patients, and hospice with high baseline proportion of institutionalized patients[b]

	Hospices with low baseline proportion of institutionalized patients		Hospices with high baseline proportion of institutionalized patients	
	Estimate (95% CI)	p-value	Estimate (95% CI)	p-value
Percent new monthly enrollees with ADRD				
Pre-FTT/debility exclusion slope [c]	0.06 (0.05, 0.08)	<.001	0.04 (0.03, 0.05)	<.001
Change during FTT/debility exclusion notice	2.84 (2.11, 3.58)	<.001	5.64 (5.06, 6.22)	<.001
Post-FTT/debility exclusion slope [c]	0.03 (-0.05, 0.1)	0.474	0.10 (0.04, 0.16)	<.001
Change during IMPACT passage	-0.24 (-1.6, 1.11)	0.725	-2.24 (-3.29, -1.19)	<.001
Change during IMPACT implementation	-2.52 (-3.9, -1.15)	<.001	-1.05 (-2.13, 0.03)	0.057
Post-IMPACT slope [c]	0.10 (0.02, 0.17)	0.01	0.09 (0.03, 0.14)	0.004
Change during TTP implementation	-0.17 (-0.86, 0.53)	0.64	0.54 (-0.01, 1.09)	0.055
Post TTP-slope [c]	0.03 (0.02, 0.05)	<.001	0 (-0.01, 0.02)	0.521
Percent monthly patient census with ADRD				
Pre-FTT/debility exclusion slope [c]	0.12 (0.11, 0.12)	<.001	0.12 (0.11, 0.13)	<.001
Change during FTT/debility exclusion notice	2.64 (2.21, 3.06)	<.001	4.29 (3.94, 4.63)	<.001
Post-FTT/debility exclusion slope [c]	0.07 (0.03, 0.11)	0.001	0.15 (0.12, 0.18)	<.001
Change during IMPACT passage	-0.74 (-1.54, 0.06)	0.07	-0.94 (-1.59, -0.28)	0.005
Change during IMPACT implementation	-1.45 (-2.26, -0.63)	<.001	-1.25 (-1.92, -0.57)	<.001
Post-IMPACT slope [c]	0.05 (0.01, 0.09)	0.013	0.04 (0.01, 0.08)	0.016
Change during TTP implementation	-0.52 (-0.92, -0.12)	0.01	-0.51 (-0.85, -0.18)	0.002
Post TTP-slope [c]	0.05 (0.04, 0.05)	<.001	0 (-0.01, 0.01)	0.801
Percent monthly care days provided to patients with ADRD				
Pre-FTT/debility exclusion slope [c]	0.13 (0.12, 0.14)	<.001	0.13 (0.13, 0.14)	<.001
Change during FTT/debility exclusion notice	2.74 (2.26, 3.22)	<.001	4.45 (4.07, 4.82)	<.001
Post-FTT/debility exclusion slope [c]	0.07 (0.03, 0.12)	0.003	0.15 (0.11, 0.18)	<.001
Change during IMPACT passage	-0.80 (-1.68, 0.08)	0.073	-0.65 (-1.36, 0.05)	0.07
Change during IMPACT implementation	-1.41 (-2.31, -0.52)	0.002	-1.24 (-1.97, -0.51)	<.001
Post-IMPACT slope [c]	0.05 (0.01, 0.1)	0.024	0.03 (0, 0.07)	0.077
Change during TTP implementation	-0.65 (-1.09, -0.2)	0.004	-0.63 (-0.99, -0.27)	<.001
Post TTP-slope [c]	0.05 (0.04, 0.06)	<.001	-0.01 (-0.01, 0)	0.217

`

[b] Baseline period defined as July 2008 – April 2013. Institutionalized patients include all patients who received hospice care in a non-home setting. Hospices with low proportion of institutionalized patients include those that had lower than median percentage (38%) of institutionalized patients in at least 90% of months during the baseline period. Conversely, hospices with high proportion of institutionalized patients include those that had higher than the median percentage (38%) of institutionalized patients in at least 90% of months during the baseline period.
[c] Slope coefficients are direct estimates of the slope during each specified time-period

© 2022 Gianattasio KZ et al. *JAMA Health Forum.*

eREFERENCES

1. Taylor DH, Østbye T, Langa KM, Weir D, Plassman BL. The Accuracy of Medicare Claims as an Epidemiological Tool: The Case of Dementia Revisited. *J Alzheimer's Dis*. 2009;17(4):807-815.

© 2022 Gianattasio KZ et al. *JAMA Health Forum.*
